# Incidence of non-communicable diseases (NCDs) in HIV patients on ART in a developing country: Case of Zimbabwe’s Chitungwiza Central Hospital—A retrospective cohort study (2010–2019)

**DOI:** 10.1371/journal.pone.0252180

**Published:** 2021-05-27

**Authors:** Alexander Cheza, Boikhutso Tlou, Danai Tavonga Zhou

**Affiliations:** 1 School of Nursing and Public Health, College of Health Sciences, University of KwaZulu-Natal, Durban, South Africa; 2 Department of Medical Laboratory Sciences, College of Health Sciences, University of Zimbabwe, Harare, Zimbabwe; University of Witwatersrand/NHLS, SOUTH AFRICA

## Abstract

**Introduction:**

The incidence of non-communicable diseases (NCDs) has been reported to be rising over the years leading up to 2010. In Zimbabwe, there are few studies done to examine the incidence of NCDs in people living with HIV (PLHIV) on anti-retroviral treatment (ART).

**Objective:**

To determine the incidence of NCDs in HIV patients on ART at the Chitungwiza Central Hospital over ten years and the associated risk factors.

**Methods:**

This was a retrospective cohort study using data from 203 patients enrolled on ART at the Chitungwiza Central Hospital between 2010 and 2019. All 500 records were considered and the selection was based on participants’ consenting to the study and their strict adherence to ART without absconding. The incidence of NCDs was determined and generalized estimating equations (GEE) were used to estimate the association between NCDs and the selected risk factors.

**Findings:**

Data collected at the study’s baseline (2010) showed that the most prevalent NCD was hypertension, found in (18/203) 8.9% of the study participants, followed by diabetes (6.9%), then followed by cardiovascular diseases (CVD) (3.9%), and the least common NCD was cancer (1.9%). Incidences of all of these NCDs showed an increasing trend as the time of follow-up progressed. The factors found to be significantly associated with the development of NCDs were gender (p = 0.002) and follow-up time (p<0.001). Geographical location was a significant risk factor as urban patients were more likely to develop hypertension as compared to the peri-urban patients (p = 0.001).

**Conclusions:**

NCDs and HIV comorbidity is common with women more likely than males to develop NCDs as they advance in age. There is need to devise targeted intervention approach to the respective NCDs and risk factors since they affect differently in relation to the demographic details of the participants.

**Recommendations:**

This paper recommends a multi-stakeholder approach to the management of NCDs, with researchers, clinicians and the government and its various arms taking a leading role.

## Introduction

Non-communicable diseases (NCDs) contribute an estimated 41 million deaths to the annual mortality rate globally and account for a total of 71% of the annual global deaths. Approximately 85% of these fatalities occur in low- and middle-income countries (LMICs) [1]. The NCDs with the leading case fatalities include cardiovascular diseases, which account for approximately 17.9 million deaths each year, followed by cancers with 9 million deaths. Respiratory diseases account for nearly 3.9 million deaths, and diabetes is responsible for 1.6 million deaths worldwide each year [1]. The persistent HIV burden predisposes HIV positive patients to NCDs due to persistent inflammation [2]. Moreover, behavioral risk factors such as tobacco smoking and alcohol use, and occupational hazards also increase the chances of PLHIV developing NCDs [3, 4].

Globally, more attention and resources have been dedicated towards dealing with HIV/AIDS, TB and malaria over the past few decades and there has been dramatic progress in patient outcomes, evidenced by improved health preparedness for these infectious diseases [5]. Despite this, in 2010 tuberculosis and malaria killed about two million people worldwide, whilst in the same year cancers killed four times as many people; about eight million [4]. This example presents the same picture across other NCDs such as heart disease, hypertension and diabetes [6]. The public health burden of NCDs in sub-Saharan Africa has been growing over the past two decades, resulting in premature deaths [7]. Sub-Saharan Africa is the region most affected by HIV/AIDS, accounting for close to 66% of the global population living with HIV/AIDS in 2017 [1, 8]. Challenges surrounding the reduction of HIV related deaths have been partly attributed to increases in NCDs. In 33 patient cohorts studied in sub-Saharan Africa, NCDs accounted for 56% of the morbidity and 40% of the mortality, and it has been shown that there is an increasing cumulative incidence of NCDs with each year of taking ART in sub-Saharan African cohorts [9]. These NCDs propel mortality in Southern Africa and all over the world. Studies in sub-Saharan Africa have shown that less than two-thirds of patients initiated on ART are on treatment two years after ART initiation [10]. A scoping review by Mudie et al. noted that NCDs are a major and growing problem in the sub-Saharan African region [11]. Many reviews have also shown that the 24-month ART retention rate is approximately 70% and the retention rate is 65% at 36 months [12].

The disease burden of NCDs in Africa has been on the rise and Zimbabwe has similarly been seeing a rise in mortality due to NCDs. Zimbabwe’s population numbers about 14 million people and it is one of the countries in sub-Saharan Africa worst affected by the HIV and AIDS epidemic [11]. Of this population, UNAIDS estimated that as at the end of 2019, 12.7% of the people aged 15 years and above in the country were living with HIV/AIDS [13]. This prevalence rate of adult HIV has been on a declining trend since the turn of the millennium, down from 27.2% in 1998 to 13.7% in 2017 [14]. The emergence of ART, has thus lowered the devastating effects of the HIV/AIDS epidemic. In terms of the adult HIV prevalence in the country, the Zimbabwe Demographic and Health Survey showed a slightly higher HIV prevalence rate in urban areas than in rural areas, and ranged by province from 13% in Harare metropolitan province to 21% in Matabeleland South province [12].

People living with the human immunodeficiency virus (PLHIV) are susceptible to opportunistic diseases owing to their weakened immune systems caused by the HIV. This resulted in numerous deaths prior to the invention of ART. However, the subsequent discovery of highly active anti-retroviral therapy (HAART) in the late 1990s led to a significant improvement of the life expectancy of patients infected with HIV [15]. Nevertheless, it is estimated that at least 36 million people die globally each year from NCDs [7]. The most prevalent NCDs which account for at least 80% of the global deaths include diabetes, cancers, cardiovascular diseases, and chronic obstructive pulmonary disease [2]. These NCDs share four common risk factors, namely hereditary and environmental factors, aging and socio-economic status, and the effects can be severe for PLHIV. Another cohort study showed that antiretroviral therapy (ART) continues to dramatically reduce rates of mortality from HIV infection in high-income countries, such that non-AIDS-related deaths exceed AIDS deaths after approximately four years of taking ART [16].

Despite the devastating effects of NCDs on PLHIV, an integrated approach focusing on LMICs remains unexplored as much evidence of NCDs is still concentrated on the high income countries [11, 17]. An integrated approach enhances the synchronization of key actors and their actions to address health needs. It is thus imperative to examine the effect of the burden of NCDs on PLHIV in LMICs, focusing on NCDs such as diabetes mellitus, hypertension, cancers, and obesity. Kansiime et al. found a 20.7% chance of developing at least one NCD in PLHIV in Uganda, whilst the most prevalent NCD was found to be hypertension [18]. The high prevalence of NCD risk factors and unrecognized and untreated hypertension represent major problems [19]. There has, however, been evidence from other studies of no association between HIV viral load and NCDs comorbidity [20]. This is consistent with other studies in the same context in South Africa, but differs from findings obtained in high income countries.

The aim of the study was to determine the incidence and the associated risk factors of NCDs in HIV patients on ART at the Chitungwiza Central Hospital (CCH). The NCDs under consideration in the study were diabetes, cardiovascular diseases (CVD), hypertension and several cancers. Such a study was important for guiding policy formulation for national public health policies in Zimbabwe and other LMICs. The study also provided empirical evidence that informed the efforts of stakeholders in the health sectors on the public health challenges requiring integrated management approaches.

## Materials and methods

The study was a retrospective analysis of data from a longitudinal hospital-based cohort. The longitudinal study design enabled the collection of data from the medical records of the study subjects over a ten-year period. The study used data from patients enrolled for ART at the Chitungwiza Central Hospital’s Opportunistic Infections Clinic (CCH OIC) in January 2010, who had not absconded from ART or died by 2019. Records were examined at the initiation point of the study and then after five and ten years. The timeframe allowed the researcher to establish the relationship between ART and the incidence of NCDs in HIV infected persons enrolled on ART at the CCH OIC, among many other factors likely to increase the incidence of NCDs in the cohort. The incidence was measured after the first five years and the results noted, and the records were then re-examined after the subsequent five years. The purpose was to determine the incidence of the various NCDs and establish whether there was an association between the demographic variables and the development of NCDs in PLHIV who were undergoing ART. Application of the longitudinal study design entailed the extraction and analysis of data at different points from the records of the patients undergoing ART after every five years.

### Study setting

The study was conducted at the CCH, a 500 bedded hospital located about 30 kilometers to the south east of Zimbabwe’s capital city, Harare. The CCH had been ISO certified since 2008. Chitungwiza had an estimated population of 1.5 million people, serving circa 400 000 patients annually. Due to high unemployment levels in Zimbabwe, many of the citizens within the catchment area of the CCH OIC were unemployed, and hence survived on informal self-help jobs such as vending for those in towns, and market gardening for the peri-urban and rural population. The catchment area of the CCH was made up of Chitungwiza town which formed the major urban area, spanning about 45 square kilometers, as well as peri-urban and rural areas covering an estimated 32 230 square kilometers. The peri-urban and rural areas served by the CCH included Beatrice, Mahusekwa, Marondera, Goromonzi, Murehwa and Nyadire [17].

### Study participants

The recruitment and selection of the participants in the study is outlined in the following section.

### Participants’ recruitment strategy

**Fig 1:**
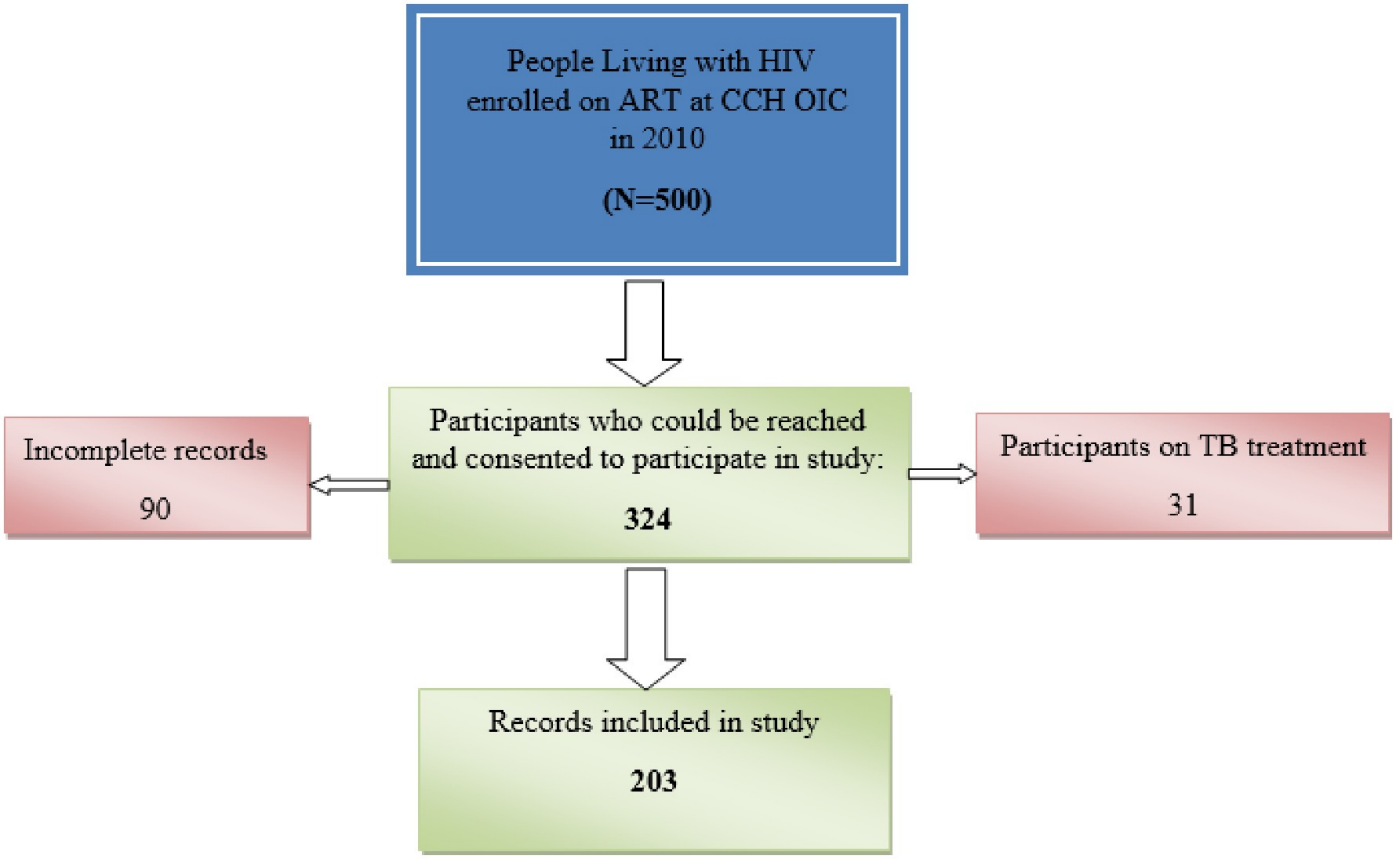
Participants’ records selection flow diagram.

All participants who were registered patients on ART at the CCH OIC were eligible for the study. Details of the participants’ selection are shown in Fig 1.

The study considered the 500 records in the register in 2010. After applying the inclusion/exclusion criteria, 203 records were selected as shown in Fig 1. Access to records was done after gatekeepers’ permission to access the patients’ records was granted by the Ministry of Health and Child Care and the CCH Administration. Consent was also sought from the participants whose records had met the inclusion criteria shown below.

### Study data

The CCH OIC was a referral clinic for several primary clinics and the following locations within the CCH catchment area were used: Chitungwiza Central, Dema, Seke North, Seke South, St Marys and Zengeza. Data was collected from the clinic’s records for the specific periods that the patients were attended to there. The following section of the paper presents the specifics of the data collected.

#### Unit(s) of analysis

The total population for the study was 500 patients who were seen at the CCH OIC in the year 2010 and who were enrolled for ART for the first time.

#### Inclusion criteria

- Patients enrolled on ART and who were in the CCH OIC data records.
- Only records for PLHIV who enrolled for ART during 2010 and continued without absconding until December 2019 were included.
- Only participants enrolled for the adult first line ART regimen at CCH OIC were included in the study.

#### Exclusion criteria

- Participants who did not consent to participate in the study were excluded.
- Individuals on TB treatment, even if they presented with any malignancy regardless of their age, were excluded from the study.
- Individuals referred for ART from outside of Zimbabwe’s primary health care system were also excluded.

### Data collection procedures

A data collection sheet was used to gather the patients’ data for analysis. The data sheet captured pertinent data from the patients’ records at CCH OIC and all of the entries were allocated study identification numbers to ensure anonymity of the patients’ records. Only patients on ART were considered. Age and gender were the demographic information captured. As the patients were assessed for the development of NCDs, geographical location and time of follow-up in the study were recorded. The NCD status of the patients was recorded at baseline and reviewed at subsequent five-year intervals to assess the progression and development of new cases.

**Fig 2:**
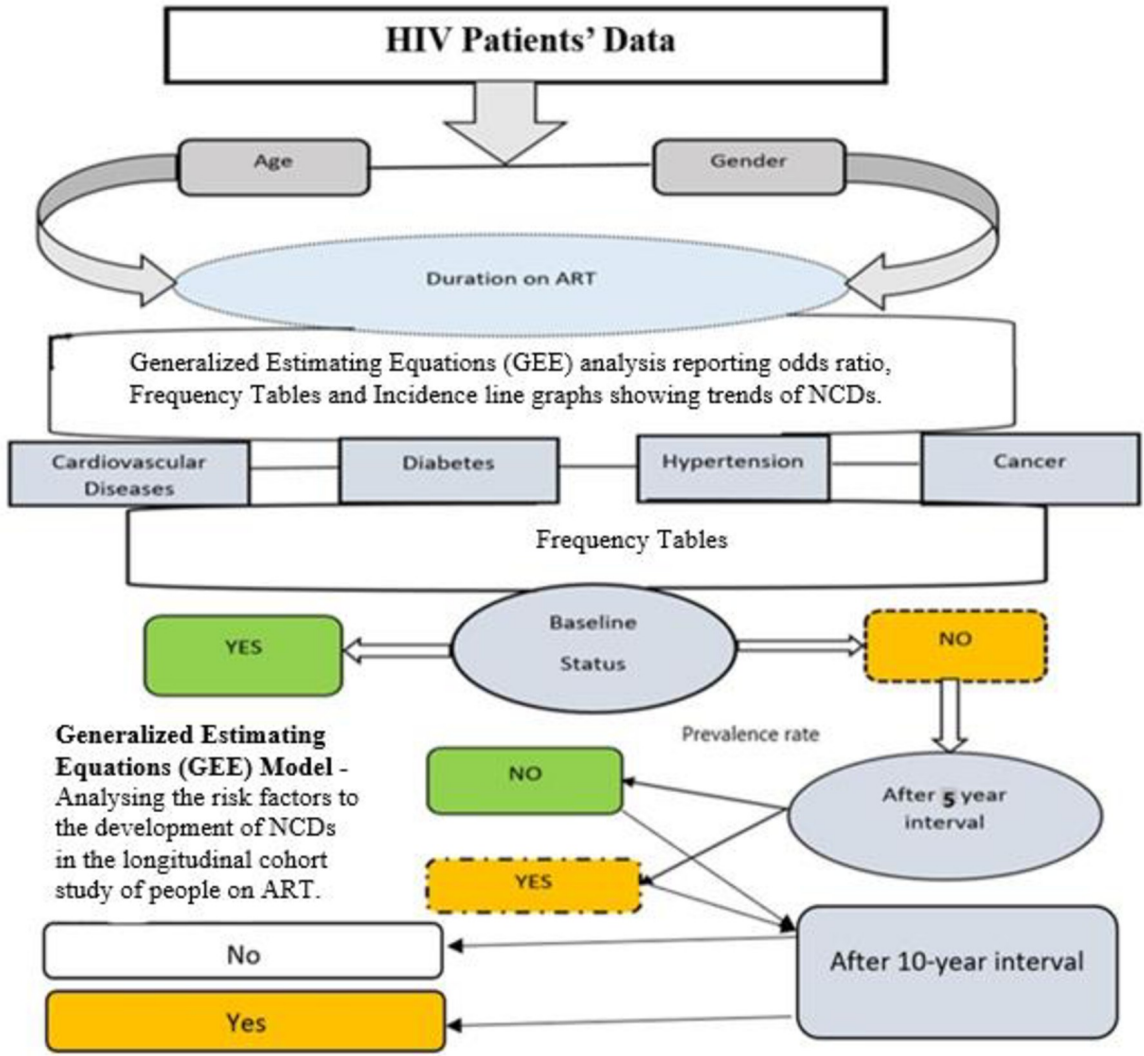
Methodology framework.

The primary aim of the study was to determine the incidence of various NCDs in PLHIV who were on ART. The study also assessed the risk factors associated with NCDs in PLHIV, with a view to ascertain if there was any association between usage of ART and susceptibility to NCDs for the selected risk factors. The patients’ records were extracted from the computerized health information department at the hospital. As shown in Fig 2, the data collected was analyzed in terms of gender, age, geographical location, and time of follow-up in the study. This analysis was guided by literature on the possibility of a causal relationship between gender, age, geographical location and time of follow-up in the study and NCDs development. The specific NCDs under consideration in this study were cardiovascular diseases, hypertension, cancer, and diabetes. All records for the 203 patients enrolled onto the study were re-examined at five-year intervals, as depicted in Fig 2 below.

#### Outcome variable

All of the response variables (cardiovascular diseases, diabetes, hypertension and cancers) were assigned binary numbers (0 = not diagnosed and 1 = diagnosed).

#### Explanatory variable

The independent variables that were assessed for their influence on NCDs were age, gender, geographical location and time of follow-up in the study.

#### Ethical consideration

Ethical approvals for the study were received from the Biomedical Research Ethics Committee of the University of KwaZulu-Natal (BE057/19) and the Medical Research Council of Zimbabwe (MRCZ/A/2441).

### Statistical analyses

Data was analyzed using Stata v13 software. Incidence rates were calculated as the new NCD diagnosed, divided by the population in each category. Frequency tables, bar graphs and pie-charts were used to summarize the demographic characteristics. The study used the Generalized Estimating Equations (GEE) approach to determine the risk factors associated with NCDs. The NCDs were also assessed for their influence on the development of other NCDs in participants already diagnosed with any NCD. All of the NCD response variables were assigned a binary number (0 = not diagnosed and 1 = diagnosed). A p-value < = 0.05 was deemed to be statistically significant. The methodology framework is summarized in Fig 2.

Fig 2 pictorially depicts the methodology adopted in the study, from initiation of the study up to the analysis of the results after the initial five-year interval and after the subsequent five-year period of the study.

## Results

**Fig 3:**
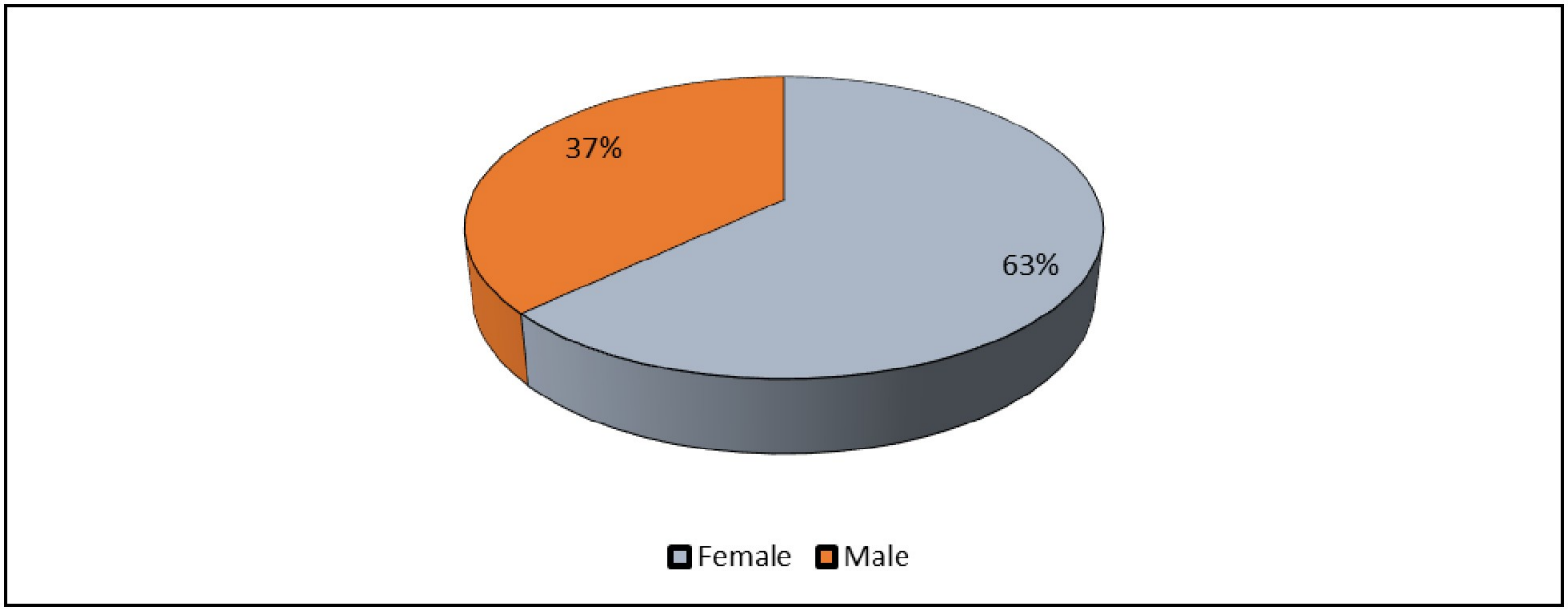
Participants’ gender.

The demographic features of the 203 participants were observed and of these 128 were females (63%), whilst 75 (37%) were males, as shown in Fig 3 below. In terms of the geographical spread of the study participants, the majority were from the urban and peri-urban areas (193 out of the 203), coming from areas such as Seke, Zengeza and St Marys, whilst only 10 came from the Dema rural area. Study participants were also spread across various age groups, which are presented later.

**Fig 4:**
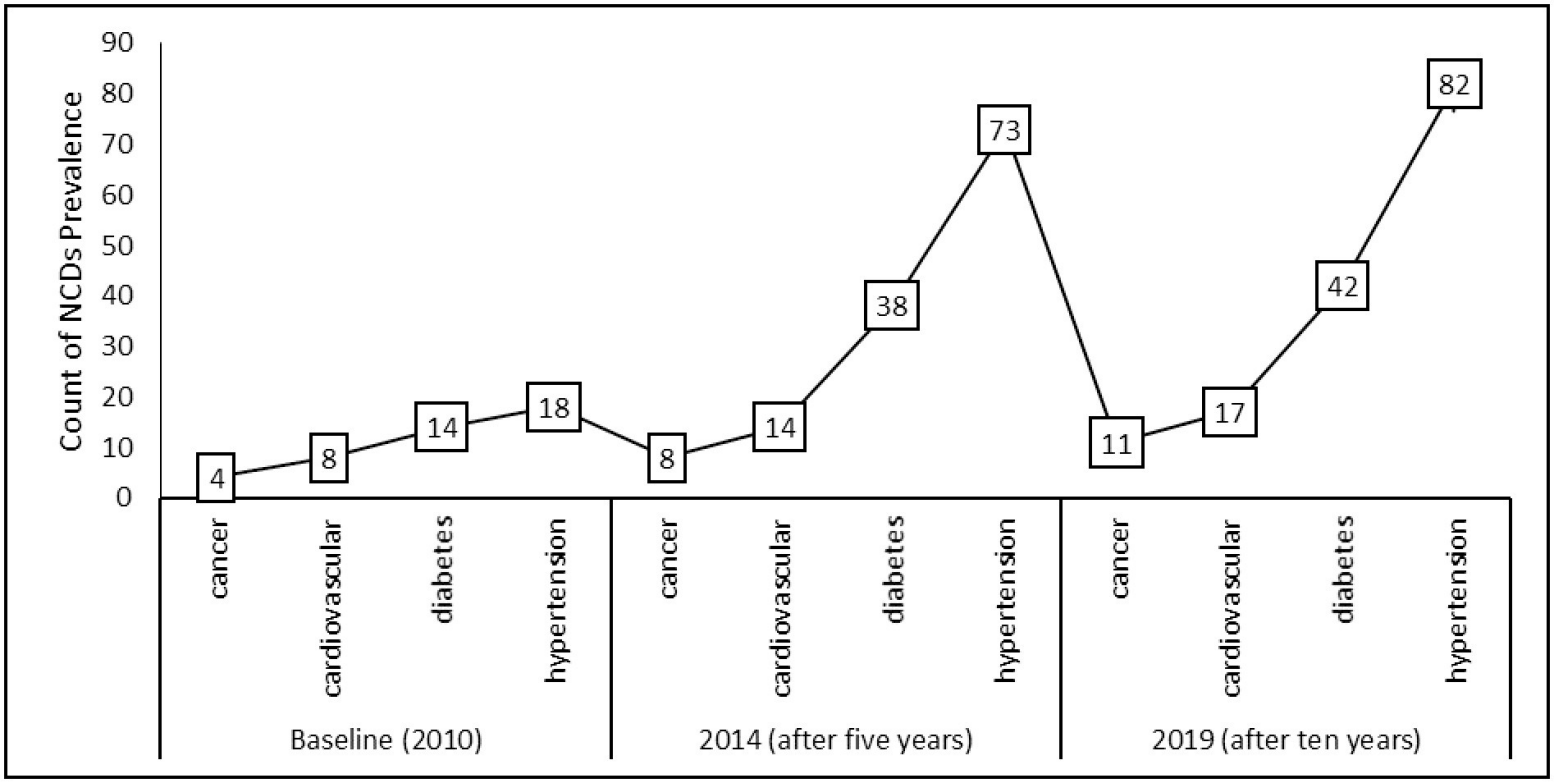
Prevalence of NCDs over time.

Of note is the fact that some participants were diagnosed with more than one NCD and the trend is presented in Fig 4.

Fig 4 summarizes the cumulative count of the patients who were diagnosed with one or more of the four NCDs during the study period. From the 203 participants at the commencement of the study, 4 (1.97%) were diagnosed with cancer, 8 (3.94%) had cardiovascular diseases, 14 (6.9%) were diabetic and 18 (8.87%) were hypertensive. All participants were followed-up after five years and after ten years to establish the incidence of NCDs. As shown in Fig 4, after the first five years hypertension was the most prevalent NCD in the 203 participants, with a prevalence rate of 35.96% (73 out of 203 participants), having increased from the baseline rate of 8.87%. The hypertension prevalence rate after the second follow-up did not increase as much as it did at the first follow-up, and the prevalence rate moderately increased to 40.39% (82 out of 203 participants). The next most prevalent NCD was diabetes, with a prevalence rate of 18.72% (38 out of 203 participants). This was a significant increase from the baseline observed prevalence of 6.9%. The cumulative prevalence of diabetes after the second follow-up also marginally increased to 20.69% (42 out of 203 participants). The next ranked prevalent NCD, as shown in Fig 4, was cardiovascular diseases with a 6.90% (14 out of 203) prevalence rate, which had nearly doubled from the baseline prevalence of 3.94%. Like the other NCDs, the prevalence slowed down in the second follow-up period. According to the data collected, the least prevalent NCD was cancer which had a baseline observation of 1.94% at commencement of the study and it doubled after five years to 3.94% prevalence. There was an almost constant increase in observed cancers during the last interval of the follow-up, with a cumulative prevalence of 5.42%.

**Fig 5:**
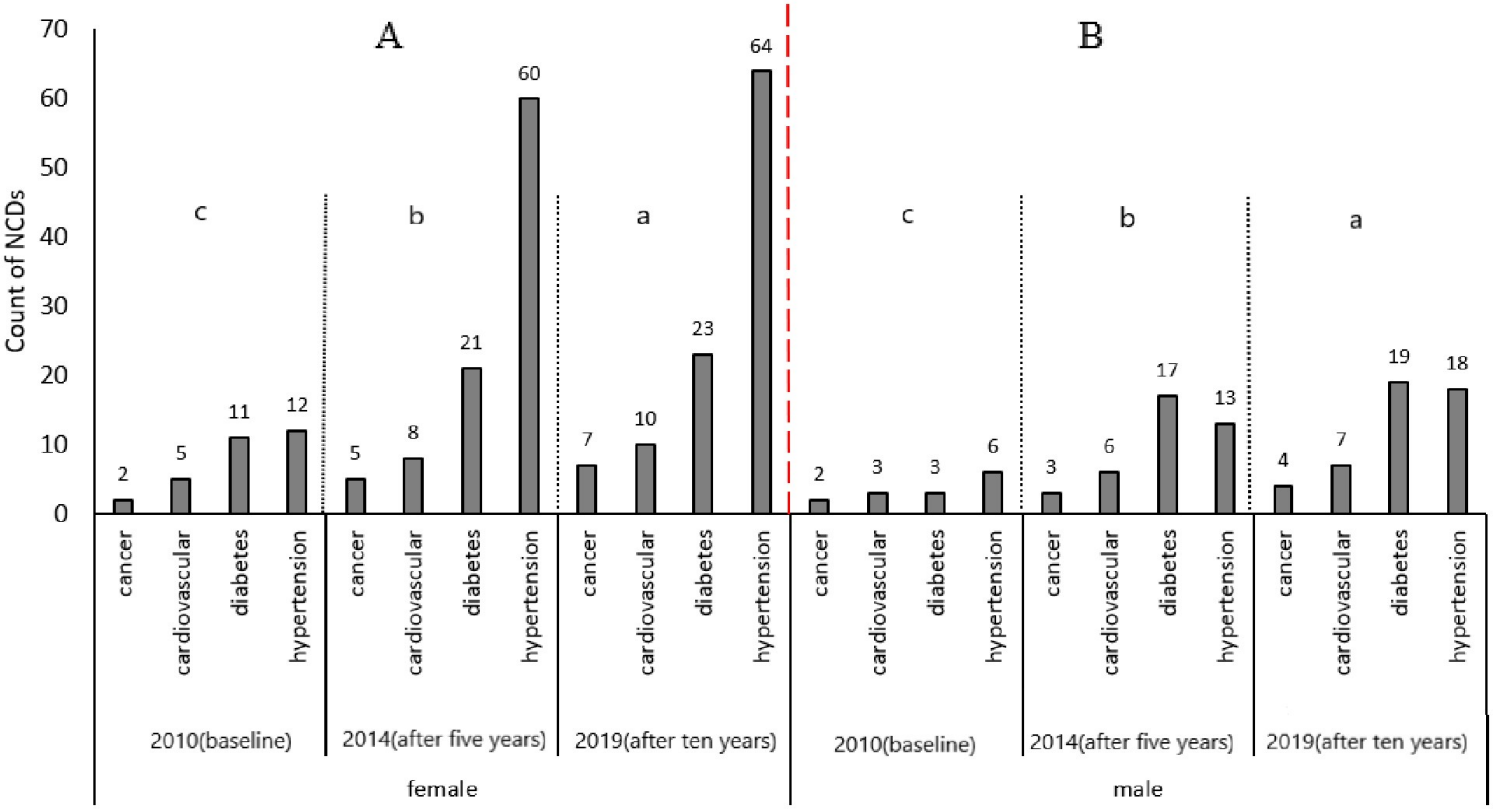
Analysis of NCD prevalence by gender, with ‘mean NCDs count separation letter’. A–Female category; B–Male category; a–Time interval after ten years; b–Time interval after five years; c–Time at baseline of the study (2010).

The study also examined the development of NCDs by gender to establish if there was any association between NCDs’ diagnosis and participants’ gender. There were 128 (63%) female participants and 75 (37%) male participants in the study. Fig 5 summarizes the results of the analysis.

As shown in Fig 5, women were more likely (p<0.001) to develop hypertension and diabetes than men, as depicted by the towering bars in Fig 5. In terms of the NCDs, Fig 5 supports the view that hypertension was the most prevalent NCD, followed by diabetes in both men and women, as the bars representing hypertension were the tallest in both females and males. In addition to the analysis of the prevalence of NCDs based on gender, Table 1 presents the incidence rates of the NCDs based on gender and age groups.

**Table 1:** Analysis of NCDs’ incidence based on gender and age.

Age group	NCDs	Follow up							
		Female				Male			
		Baseline	Five-year Interval	Ten-year Interval	Total	Baseline	Five-year Interval	Ten-year Interval	Total
**<40 years**	Cancer	0	2/45	2/45	**4/45**	0	2/8	2/8	**4/8**
	Cardiovascular	2/45	0	0	**2/45**	1/8	1/8	1/8	**3/8**
	Diabetes	1/45	5/45	5/45	**11/45**	0	0	0	**0**
	Hypertension	0	14/45	14/45	**28/45**	1/8	0	0	**1/8**
**40–55 years**	Cancer	0	3/98	4/98	**7/98**	1/40	0	1/40	**2/40**
	Cardiovascular	1/98	4/98	4/98	**9/98**	0	3/40	3/40	**6/40**
	Diabetes	4/98	8/98	8/98	**20/98**	2/40	9/40	9/40	**20/40**
	Hypertension	3/98	29/98	30/98	**62/98**	1/40	5/40	6/40	**12/40**
**>55 years**	Cancer	2/85	0	1/85	**3/85**	1/53	1/53	1/53	**3/53**
	Cardiovascular	2/85	4/85	6/85	**12/85**	2/53	2/53	3/53	**7/53**
	Diabetes	6/85	8/85	10/85	**24/85**	1/53	8/53	10/53	**19/53**
	Hypertension	9/85	17/85	20/85	**46/85**	4/53	8/53	12/53	**24/53**

**Fig 6:**
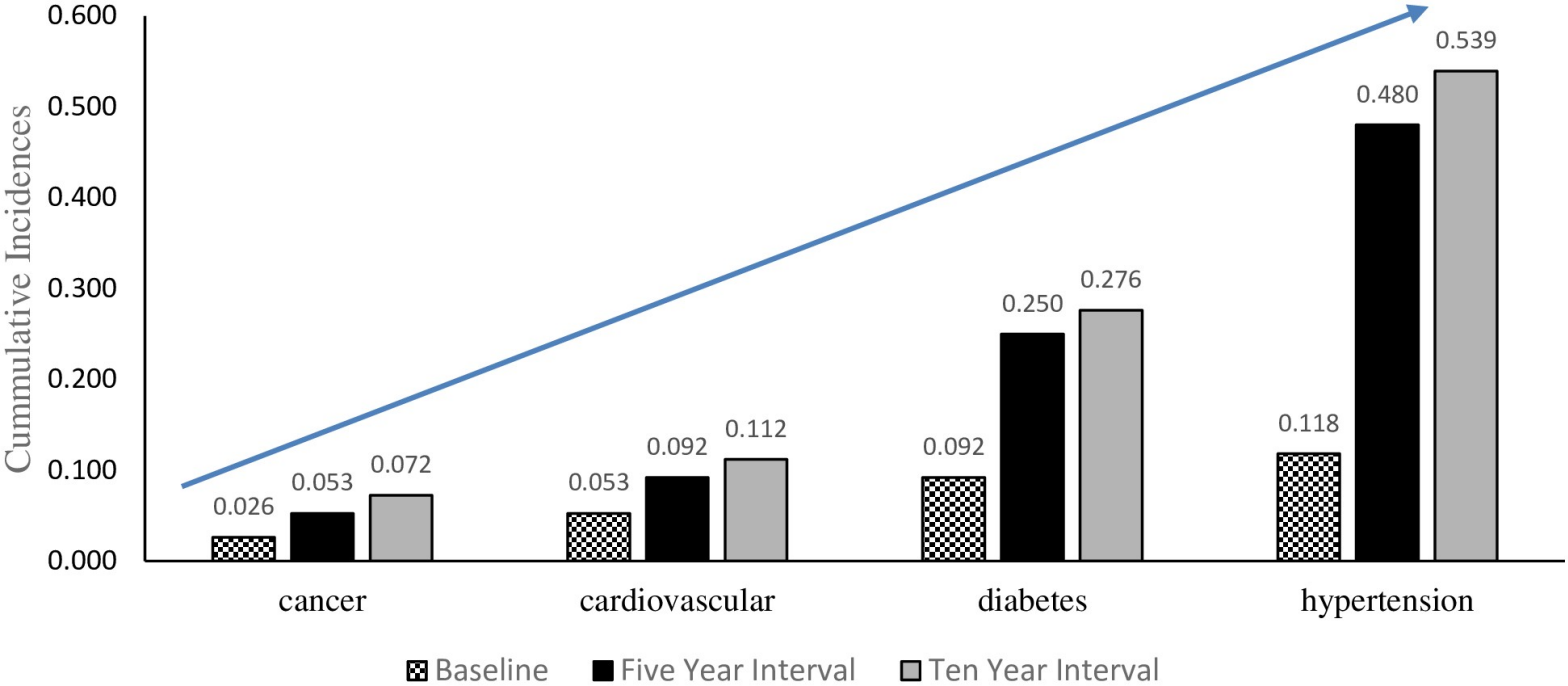
Incidence of NCDs over time.

Table 1 supports the views presented in Fig 5. The incidence of cancer in both males and females aged less than 40 years was small, although relatively higher in males (2/8). Similar trends in incidences of cancer were observed in both males and females in the other age groups. The incidence of cardiovascular diseases (CVDs) showed a general increase over time as the age of the participants also increased. The incidence of diabetes also increased as age increased in both males and females over the study period, with the highest incidences recorded in participants aged above 55 years in both males and females. The incidence of hypertension also increased in both male and female participants as age increased. The highest incidence of hypertension was in participants older than 55 years for both male and female participants in all of the follow-ups. The incidences of the NCDs in both males and females presented in Table 1 are summarized and synthesized in Fig 6, which shows the general trend for all four NCDs under study over the study’s timeframe.

As shown in Fig 6, the most common NCD was hypertension, which increased over time, followed by diabetes, CVDs and cancer in descending order.

Table 2 shows that participants who resided in urban areas were 3.287 times more likely to develop hypertension when compared to those who resided in peri-urban locations (p-value = 0.001). However, participants from rural areas were about 30% less likely to develop hypertension as compared to peri-urban participants. Similar observations were obtained for diabetes. Across all of the NCDs, the urban participants were more likely to develop NCDs than the peri-urban participants. Results in Table 2 revealed that patients aged > = 45 years were 1.387 times more likely to develop various NCDs when compared to patients aged <25 years. This trend was also shown in the odds of developing hypertension and diabetes, which were 2.496 and 1.318 times respectively more likely to develop in patients aged greater than or equal to 45 years in comparison to the patients less than 25 years of age.

**Table 2:** Odds ratios for risk factors associated with NCDs using generalized estimating equations.

		Hypertension			CVD			Diabetes			NCDs		
Explanatory variables	Categories of Explanatory Variables	Odds Ratio	95% Confidence Interval	p-value	Odds Ratio	95% Confidence Interval	p-value	Odds Ratio	95% Confidence Interval	p-value	Odds Ratio	95% Confidence Interval	p-value
**Geographic Location**	Rural	0.701	0.109–4.530	0.709	4.401	0.755–25.655	0.099	0.776	0.098–6.136	0.810	1.474	0.420–5.175	0.545
	Urban	3.287	1.626–6.644	0.001	0.641	0.258–1.591	0.337	1.905	0.883–4.111	0.101	1.739	0.983–3.077	0.057
	Peri-urban	1.000			1.000			1.000			1.000		
**Age**	> = 45 years	2.496	0.219–28.442	0.461	0.584	0.069–4.923	0.621	1.318	0.112–15.539	0.826	1.387	0.273–7.060	0.693
	35–44	0.995	0.082–12.108	0.997	0.210	0.016–2.827	0.240	0.373	0.028–4.893	0.453	0.500	0.094–2.659	0.416
	25–34	1.856	0.183–18.814	0.601	0.486	0.090–2.625	0.402	0.448	0.102–1.963	0.287	1.051	0.253–4.370	0.945
	<25	1.000			1.000			1.000			1.000		
**Gender**	Female	3.623	2.076–6.324	<0.001	0.877	0.357–2.151	0.774	1.402	0.761–2.583	0.278	2.097	1.315–3.343	0.002
	Male	1.000			1.000			1.000			1.000		
**Time Interval**	Ten years	9.449	4.612–19.360	<0.001	2.873	1.069–7.726	0.036	6.335	3.098–12.953	<0.001	8.160	4.738–14.055	<0.001
	Five years	7.860	3.985–15.502	<0.001	2.107	0.796–5.579	0.133	5.382	2.733–10.598	<0.001	7.268	4.339–12.174	<0.001
	Baseline	1.000			1.000			1.000			1.000		
**Diabetes**	Yes	0.472	0.252–0.884	0.019	0.399	0.106–1.501	0.174						
	No	1.000			1.000								
**CVDs**	Yes	1.748	0.704–4.340	0.229				0.408	0.106–1.570	0.192			
	No	1.000						1.000					
**Cancer**	Yes	0.215	0.050–0.925	0.039	1.293	0.135–12.335	0.824	0.868	0.153–4.919	0.873			
	No	1.000			1.000			1.000					
**Hypertension**	Yes				1.876	0.793–4.438	0.152	0.441	0.228–0.853	0.015			
	No				1.000			1.000					

Moreover, female patients showed a consistent trend across all of the NCDs to be about two times more likely to develop NCDs compared to male patients (p-value = 0.002). Female patients were 3.623 times more likely to develop hypertension as compared to male patients (p-value < 0.001). Females were 1.402 times more likely to be diabetic when compared to male patients. However, CVDs did not follow the same pattern and females were about 10% less likely to develop the condition in comparison to the male participants.

Results revealed that as follow-up time increased, the odds of developing NCDs also increased. Patients were eight times more likely to be diagnosed with at least one NCD after ten years of follow-up when compared to their baseline results (p-value < 0.001). At five-year intervals, the patients were seven times more likely to have developed an NCD as compared to their baseline results (p-value <0.001). This trend occurred across all of the individual NCDs, showing more likelihood of developing the conditions over time when comparing the incidence of these diseases in these same patients at baseline. The odds of becoming hypertensive increased to about 9 times more likely after 10 years (p-value < 0.001), whilst participants were 2.873 times more likely to develop CVDs (p-value = 0.036) and about 6 times more likely to be diabetic after 10 years (p-value < 0.001).

In addition, the results showed that patients who had already been diagnosed with diabetes had a lesser chance of developing hypertension (p-value = 0.019), and this was also true for the patients who were hypertensive at baseline as they were less likely to develop diabetes (p-value = 0.015). The CVD patients were seen to be 1.748 times more likely to be hypertensive as compared to the non-CVD patients. Cancer patients showed that they were 1.293 times more likely to develop CVD as compared to the patients without cancer. However, cancer patients were about 80% less likely to develop hypertension as compared to the non-cancer patients (p-value = 0.039). Nevertheless, we could not estimate the risk factors associated with cancer due to the low number of cancer cases recorded in the study.

## Discussion

We observed an increase in incidence across all four NCDs under study, with differing magnitudes for each condition. Participants were more susceptible to hypertension, followed by diabetes, and although the incidence was the lowest for cancer, there is a need to respond equally to this NCD due to the fatality associated with the condition. We concluded, based on the study’s results that the likelihood of being diagnosed with hypertension increased as follow-up time increased, with the odds increasing from 7.860 to 9.449. This increase in incidence could not be explained merely by analyzing the calculated incidence rates shown in Fig 4, hence we analyzed the risk factors associated with the development of the various NCDs. We determined the odds of one developing an NCD against each risk factor and we concluded that participants who resided in urban areas were more likely to be diagnosed with NCDs in comparison to those in peri-urban locations.

Based on the GEE model, our results showed that PLHIV in rural areas were about 1.5 times more likely to develop at least one NCD when compared to those residing in peri-urban settings. These findings confirmed results obtained by Kavishe et al. [19], who studied and found the prevalence of hypertension to be higher in rural areas than in urban areas of Tanzania and Uganda. There are several causes for such high incidences of NCDs in rural areas which may include but are not limited to lifestyle differences and economic capacity to seek medical assistance. Participants aged 45 years and above were more susceptible to developing NCDs when compared to participants aged below 25. As people got older they became more prone to different NCDs like hypertension and geriatric diabetes. This was consistent with several studies done in several LMICs where older participants in the cohorts were more likely to be diagnosed with NCDs [15–20].

Our findings revealed that the odds of being diagnosed with at least one NCD increased as the follow-up time interval increased. Since participants were enrolled onto ART at the start of the cohort study, the increase in follow-up time intervals was equivalent to the duration on ART. Therefore, participants were more likely to be diagnosed with NCDs as follow-up times increased, as a result of the increasing ages of the respective participants. The results were consistent with findings obtained in other longitudinal studies in other LMICs [4, 7, 17, 18]. However, our results showed that women were more likely to develop NCDs than men, and these results could not be confirmed from literature as other studies revealed that males were more likely to develop NCDs [18]. The discrepancy could be attributable to the difference in study settings and the gender composition of the studies’ participants; in our study 63% were females. In addition, our findings revealed that diagnosis with hypertension increased the odds of developing CVDs, and participants with CVDs were also more likely to develop cancer and become hypertensive. These results showed a cross-morbidity between hypertension and CVDs.

Overall, the burden of NCDs was not only prevalent in Zimbabwe but was a common public health challenge in the sub-Saharan Africa region, as observed in a scoping review by Mudie et al. [11]. Our findings revealed that the most prevalent NCD was hypertension and that diabetes was the second most prevalent NCD; results which were confirmed by other studies from LMICs [18–20]. This was likely due to hypertension and diabetes being common comorbid NCDs, which implied that they were more likely to affect the same patients. One of the least prevalent NCDs was cancer in the scoping review by Mudie et al. [11], a result which was also confirmed by this study. Despite the cancer incidence and prevalence being reported as low in our study and in other studies, it must be given equally high priority in management because of the difficulty in managing the condition and the high fatality rates associated with cancer [2–5].

The strengths of this study included the elimination of data collection bias through the use of clinicians without vested interests in the study results besides academic knowledge generation. The data was also collected over a ten-year timeframe, implying that the time frame was long enough to allow for the development of NCDs up to levels when the conditions could be observable. Thus, the longitudinal nature of the data collected allowed the results to be detectable. Conversely, the major weaknesses of the study included the unavailability of HIV related data about the participants, such as CD4 cell counts and the exclusion of traditional risk factors for NCD development such as tobacco use and alcohol use, among others. The study also regarded the time of the participants’ diagnosis as the time of developing the NCD condition. Despite the significant contribution of this study, our results cannot be generalized since we only conducted the study at one facility.

Chitungwiza has a high prevalence of hypertension and diabetes in PLHIV and therefore there is a need for a multiple stakeholder approach to managing these NCDs [3, 4, 6, 15]. Despite the relatively lower prevalence and incidence in cancer and cardiovascular diseases, there is a need for the Zimbabwean government to increase resources and efforts in managing cancer in the population living with HIV and AIDS [3, 4].

## Conclusions

All of the NCDs, namely hypertension, diabetes, cancer, and CVDs progressed constantly over time, as the prevalence rates after the first five years were almost like the prevalence rates after the subsequent five years. This increase in NCDs was supported by the results of the GEE. It was determined that more effort is needed for the management of the least prevalent NCDs (cancers and CVDs) due to their high mortality rates, as reported in literature, since cancer is now a leading killer globally.

## Declarations

**Consent for publication**: Written consent to participate in the study was sought from each participant and only records from individuals living at the time of this study were used. Participants consented to the study’s findings being published without any jeopardy to their image.

## Supporting information

S1 Dataset (XLS)

## Abbreviations

AIDS: Acquired Immune Deficiency Syndrome
ART: Anti-Retro Viral Therapy
CCH: Chitungwiza Central Hospital
CVD: Cardiovascular Disease
HIV: Human Immunodeficiency Virus
LMICs: Low- to Medium- Income Countries
NCDs: Non-Communicable Diseases
OI: Opportunistic Infections
PLHIV: People Living with Human Immunodeficiency Virus
